# Special Issue “Molecular Mechanisms and Therapies of Pancreatic Cancer: 2nd Edition”

**DOI:** 10.3390/ijms27198735

**Published:** 2026-09-30

**Authors:** Claudia Vilardo, Annalisa Di Domenico, Donatella Delle Cave

**Affiliations:** Institute of Genetics and Biophysics ‘Adriano Buzzati-Traverso’ (IGB), CNR, 80131 Naples, Italy; claudia.vilardo@igb.cnr.it (C.V.); annalisa.didomenico@igb.cnr.it (A.D.D.)

Pancreatic ductal adenocarcinoma (PDAC) is the predominant histological form of pancreatic cancer and remains one of the most aggressive and therapeutically challenging malignancies [1,2,3,4]. To date, surgical resection remains the only potentially curative treatment; however, approximately 85% of patients present with advanced, unresectable disease [5,6,7,8]. Moreover, postoperative outcomes remain poor, with >80% of resected patients experiencing disease progression within five years [9,10,11,12]. The presence of occult micrometastatic lesions at the time of resection, together with the highly aggressive and heterogeneous nature of PDAC, represents a major determinant of treatment failure [13,14,15,16]. Although neoadjuvant and adjuvant chemotherapy can improve outcomes in selected patients, their overall survival benefit remains limited, and durable responses are uncommon [17,18]. Consequently, the 5-year survival rate of patients with PDAC remains approximately 13%, emphasizing the urgent need for more effective therapeutic approaches [19,20,21]. The limited efficacy of conventional and targeted therapies reflects not only the genetic heterogeneity of PDAC cells but also their complex interaction with the tumor microenvironment (TME) [22,23,24]. In particular, the dense desmoplastic stroma promotes hypoxia, immune suppression, and therapeutic resistance [25,26]. Thus, PDAC is increasingly recognized as a dynamic ecosystem in which genetic, metabolic, stromal, and immune alterations are deeply interconnected [27,28]. Beyond the intrinsic molecular heterogeneity of PDAC, several environmental, metabolic, and genetic factors contribute to its development and progression. Among established risk factors, chronic pancreatitis, obesity, diabetes, and inherited genetic predisposition have been extensively investigated, with increasing evidence linking metabolic disorders to PDAC development and progression [29,30,31,32]. In this context, metformin (Met), the first-line therapy for type 2 diabetes, has attracted considerable interest for its potential antitumor activity [33,34,35,36]. Epidemiological and preclinical evidence suggests that Met may influence pancreatic cancer risk and disease outcome, extending its potential relevance beyond glucose control [37,38]. More recently, Cristovão and colleagues investigated the effects of Met in combination with sodium–glucose co-transporter 2 (SGLT2) inhibitors, including dapagliflozin and canagliflozin [39]. SGLT2 is the major renal glucose transporter, and its inhibition promotes urinary glucose excretion through an insulin-independent mechanism. Beyond glucose control, SGLT2 inhibitors have emerged as potential modulators of cancer metabolism. The authors showed that both SGLT2 inhibitors and Met exerted cytotoxic and pro-apoptotic effects in PDAC cells, which were further enhanced by combination treatment and that were independent of glucose uptake and involved PI3K- and JNK-dependent signaling. These findings, obtained in PDAC cell models, provide preclinical evidence supporting further investigation of antidiabetic drug combinations as a potential drug-repurposing strategy for PDAC. A major theme emerging from the contributions to this Special Issue is the metabolic plasticity of PDAC and its relevance to tumor adaptation and therapeutic response [40,41,42,43]. Cancer cells must continuously adapt their metabolism to support biomass production, ATP generation, redox homeostasis, and survival under conditions of hypoxia and nutrient deprivation [43,44,45,46,47]. In PDAC, these challenges are particularly pronounced because the extensive fibrotic stroma restricts vascular perfusion and creates a metabolically hostile environment. Consequently, PDAC cells undergo extensive metabolic rewiring, involving glucose, amino acid, lipid, nucleotide, and mitochondrial metabolism, as well as nutrient scavenging mechanisms such as autophagy and macropinocytosis. Importantly, metabolic reprogramming in PDAC is not uniform across tumor cells but can give rise to distinct metabolic phenotypes that adapt differently to the nutrient and oxygen gradients within the TME [48,49]. Lactate, traditionally considered an end product of aerobic glycolysis, can instead serve as an alternative carbon and energy source for tumor cells, particularly under nutrient-deprived conditions [50,51]. Baiskhanova and colleagues identified the transmembrane protease serine 11B (TMPRSS11B) as a regulator of lactate metabolism and phenotypic heterogeneity in PDAC. The authors showed that TMPRSS11B modulates lactate transport by regulating basigin (BSG), a chaperone required for the membrane localization and activity of the lactate transporter SLC16A1/MCT1. TMPRSS11B expression reduced BSG availability and consequently limited SLC16A1-mediated lactate uptake, whereas TMPRSS11B depletion increased BSG levels and enhanced lactate uptake. Enhanced lactate utilization was associated with increased proliferation, stemness, colony-forming capacity, and chemoresistance. Consistently, PDAC tissues with low TMPRSS11B and high BSG/SLC16A1 expression displayed increased expression of the stemness marker KLF4. Overall, these findings from cellular and tissue-based analyses suggest that the TMPRSS11B–BSG–SLC16A1 axis may contribute to metabolic and phenotypic heterogeneity in PDAC by regulating lactate utilization and supporting a stem-like, therapy-resistant phenotype [52]. A second major theme addressed by the Special Issue is the identification of biomarkers capable of capturing PDAC heterogeneity and improving disease monitoring and patient stratification. Given the poor prognosis and limited therapeutic response associated with PDAC, increasing efforts have focused on identifying novel biomarkers that may enable earlier disease detection, improve monitoring of treatment response and recurrence, and inform therapeutic decision-making. The contributions discussed below encompass circulating biomarkers, epigenetic alterations, and tissue-based markers, highlighting how different biological layers can provide complementary information for patient characterization and clinical management [53,54,55,56]. The serum tumor markers currently used in clinical practice for pancreatic cancer include carbohydrate Antigen 19-9 (CA19-9) [57,58,59], carcinoembryonic antigen (CEA), duke pancreatic monoclonal antigen type 2 (DUPAN-2) and s-pancreas-1 antigen (SPan-1) [60,61,62,63,64]. However, these biomarkers are generally evaluated in combination because of their limited sensitivity and specificity [62]. In recent years, circulating cell-free DNA (cfDNA) and mutation-specific circulating cell-free tumor DNA (ctDNA) have emerged as promising biomarkers for clinical and diagnostic applications. cfDNA can be detected in both plasma and urine samples, and elevated levels have been associated with tumor burden and treatment response, as cfDNA is largely released from necrotic and apoptotic cells [63,64,65,66]. Furthermore, ctDNA can reflect the genetic and epigenetic alterations present in the primary tumor, potentially enabling the detection and molecular characterization of tumor-derived DNA. A small-scale preliminary study identified 124 differentially methylated CpG sites located within intergenic regions of plasma cfDNA from patients with PDAC, suggesting that ctDNA methylation profiles may represent promising biomarkers for PDAC. Moreover, this observation is consistent with several studies showing that pancreatic cancer cells actively regulate DNA methylation to silence tumor suppressor genes, activate oncogenic pathways, and promote genomic instability [67,68]. For instance, low TGFB2 promoter methylation was observed in patients with PDAC and was associated with elevated TGFβ2 levels and increased expression of its downstream target IL-6 [69]. Interestingly, young PDAC patients with high *TGFB2* promoter methylation, as well as low TGFβ2 and IL6 levels, exhibited better overall survival (OS), highlighting the potential age- and isoform-specific prognostic relevance of TGFβ2. Notably, Trabedersen (OT-101), an antisense oligodeoxynucleotide targeting TGFB2 mRNA, was evaluated in a Phase I/II clinical study in patients with PDAC, in which improved overall survival was reported [69]. Although these findings provide clinical evidence supporting further investigation of TGFB2-targeted approaches, they require confirmation in larger and appropriately controlled clinical studies. Precision medicine represents a further important perspective emerging from the contributions to this Special Issue. By linking molecular features with treatment response, recurrence, and patient outcome, these contributions illustrate how biomarker-driven approaches may support the selection and optimization of therapeutic strategies in PDAC. The pronounced heterogeneity of pancreatic cancer contributes to variability in treatment response and represents a major barrier to effective therapy. Therefore, there is an increasing need to tailor therapeutic strategies according to the underlying disease biology and genetic profile of individual patients. Key advances towards precision medicine include the identification of factors predictive of disease susceptibility and clinical course, the development of personalized therapies, and the use of biomarkers to monitor treatment response. Among tissue-based biomarkers, mucins have attracted particular interest because their altered expression and glycosylation patterns are closely associated with PDAC biology and disease progression. In particular, mucin 5 AC (MUC5AC) is frequently overexpressed in pancreatic cancer and has been investigated as a potential marker of tumor aggressiveness, therapeutic response, and disease recurrence. Manne et al. demonstrated that expression of the heavily glycosylated mature isoform of MUC5AC was associated with poor response to neoadjuvant therapy (NAT) and a high risk of recurrence in PDAC patients undergoing curative resection [70]. Interestingly, MUC5AC was also detected in different metastatic lesions, where its levels were higher than those observed in primary tumors, further supporting its association with tumor aggressiveness and progression. These findings suggest that MUC5AC expression may have potential value for identifying patients at increased risk of poor response and recurrence, although its utility for guiding the duration of NAT or the timing of surgery requires prospective clinical validation. Another promising approach to optimize therapeutic efficacy involves rational drug design aimed at enhancing drug delivery and tumor cytotoxicity [71,72,73,74]. Imipridones are a novel class of anticancer compounds that have shown promising results in clinical trials for the treatment of several solid and hematological malignancies and were recently approved by the FDA for the treatment of recurrent H3 K27M-mutant diffuse midline glioma [75]. Second-generation imipridones with increased potency have been identified and characterized in recent years. In addition, additional structural modifications, such as the incorporation of N-methylaminopropargyl and ferrocenyl/phenyltriazolyl residues, have been shown to further enhance the efficacy of these compounds in overcoming treatment resistance. In vitro studies have shown that these compounds can induce profound, and in some experimental settings complete, tumor cell killing at relatively low concentrations. However, their cytotoxic effects on non-tumor cells may narrow the therapeutic window, and their therapeutic potential in PDAC remains to be established in appropriate preclinical models and clinical studies. Combination therapies may enable effective tumor suppression at reduced doses, thereby limiting off-target toxicity. The current first-line chemotherapy regimens for PDAC include FOLFIRINOX (a combination of 5-fluorouracil, leucovorin, irinotecan, and oxaliplatin) and Gemcitabine/nab-Paclitaxel [76]. As individual agents, these drugs have shown limited efficacy in clinical studies. Similar results have been obtained in clinical trials evaluating another promising class of anticancer agents, the FAK inhibitors (FAKi) [77,78]. Focal adhesion kinase (FAK), a non-receptor tyrosine kinase, is considered a crucial regulator of several tumor-related processes, including migration, angiogenesis, invasion, stemness and cancer cell survival. Several FAKi have been tested for various types of cancer, including pancreatic cancer, although they have shown variable and generally moderate clinical efficacy. Nevertheless, recent preclinical studies have demonstrated more promising antitumor activity when FAKi are combined with chemotherapy, immune checkpoint inhibitors, radiotherapy, or virotherapy, with evidence of overcoming treatment resistance and enhancing antitumor immune responses. For example, notable results have been obtained using FAKi in combination with CVB3 PD-H, an oncolytic virus that selectively infects and kills tumor cells, thereby inducing a strong antitumor immune response [78]. In vitro co-treatment of pancreatic cancer cells with specific optimized concentrations of FAKi and CVB3 PD-H produced synergistic and additive antitumor effects. Finally, one of the major challenges in precision oncology is the modulation of the tumor microenvironment (TME), which represents a dense and immunosuppressive environment that contributes to drug resistance. The PDAC TME comprises a mixture of stromal cells, immune cells, and rigid extracellular matrix (ECM) that supports cancer cell growth and dissemination [46]. Cancer-associated fibroblasts (CAFs) and activated pancreatic stellate cells (PSCs) are well known to interact with pancreatic cancer cells and promote their proliferation. Several studies have demonstrated that disrupting this cross-talk through the modulation of stromal receptor expression, cytokine secretion, cell reprogramming and release of metabolite-rich extracellular vesicles can exert antifibrotic effects and successfully reduce cancer aggressiveness [79,80]. Pancreatic cancer is considered a “cold tumor” and is often poorly responsive to immunotherapies, partly because of the presence of immunosuppressive cells that impede the infiltration of NK and CD8+ T lymphocytes. NK and CD8+ cells are critical components of the antitumor immune response; consequently, mechanisms of cancer immune evasion include reduced immune-cell infiltration and impaired cytotoxic activity. Efforts to overcome immune evasion have been challenged by substantial variability in the immune landscape among patients. In the context of personalized medicine, Fanijavadi and colleagues proposed a “Triple NK Cell Biomarker Approach” to predict immunotherapy outcome. This approach considers three major factors that influence NK-cell immunosurveillance: tumor features, TME changes, and NK cell phenotype profiling [77]. Along the same line, innovative strategies have been proposed to enhance NK cell activity, including CAR NK cells, NK cell engagers, NK cell-based aptamers and vaccines. Remarkably, microbiome imbalances have also been implicated in PDAC progression, chemoresistance and adverse side effects, particularly through their impact on NK cell function [81]. Integrating microbiome composition with NK cell profiles may provide valuable biomarkers for predicting treatment response and toxicity, while microbiome-targeted strategies may represent a potential avenue for modulating NK-cell activity and therapeutic response, although their clinical relevance remains to be determined. In conclusion, the contributions to this Special Issue collectively highlight that PDAC should be viewed as a dynamic and heterogeneous ecosystem in which tumor-intrinsic programs are continuously shaped by interactions with the surrounding microenvironment [82,83,84,85]. Across the studies discussed, metabolic plasticity, epigenetic regulation, extracellular matrix remodeling, immune dysfunction, and microbiome alterations emerge as interconnected processes rather than independent determinants of disease progression and therapeutic resistance. Importantly, this interconnectedness provides a framework for linking mechanistic alterations to clinically relevant features, including tumor heterogeneity, treatment response, disease recurrence, and patient outcome. Integrating molecular characteristics of cancer cells with microenvironmental, immune, and metabolic information may therefore improve biomarker development and patient stratification while providing a rationale for more context-specific therapeutic combinations [86,87,88]. Future progress will depend on the ability to capture this biological complexity at increasingly high resolution and in clinically relevant models [89,90]. The integration of multi-omics profiling with liquid biopsy, spatial and single-cell technologies, and patient-derived models may help define dynamic tumor states, identify vulnerabilities that emerge within specific tumor–microenvironment contexts, and determine which patients are most likely to benefit from therapeutic strategies. At the same time, translating these insights into clinical practice will require validation of candidate biomarkers and therapeutic approaches in well-designed prospective studies. Ultimately, a systems-level understanding of PDAC may help bridge the gap between mechanistic discoveries and precision oncology, supporting the development of more effective and durable strategies for disease detection, patient stratification, and treatment.
